# Investigating CHRNA5, CHRNA3, and CHRNB4 variants in the genetic landscape of substance use disorder in Jordan

**DOI:** 10.1186/s12888-024-05898-7

**Published:** 2024-06-11

**Authors:** Laith AL-Eitan, Mohammad Shatnawi, Mansour Alghamdi

**Affiliations:** 1 Department of Biotechnology and Genetic Engineering, Jordan University of Science and Technology, Irbid, 22110 Jordan; 2 Department of Anatomy, College of Medicine, King Khalid University, Abha, 62529 Saudi Arabia; 3 Genomics and Personalized Medicine Unit, College of Medicine, King Khalid University, Abha, 62529 Saudi Arabia

**Keywords:** Nicotinic receptor genes, *CHRNA5*, *CHRNA3*, *CHRNB4*, Substance Use Disorder

## Abstract

**Background:**

Substance use disorder (SUD) is a complex illness that can be attributed to the interaction between environmental and genetic factors. The nicotinic receptor gene cluster on chromosome 15 has a plausible association with SUD, particularly with nicotine dependence.

**Methods:**

This study investigated 15 SNPs within the CHRNA5, CHRNA3, and CHRNB4 genes. Sequencing was used for genotyping 495 Jordanian males with SUD and 497 controls matched for age, gender, and descent.

**Results:**

Our findings revealed that none of the tested alleles or genotypes were correlated with SUD. However, our analysis suggests that the route of substance use was linked to rs1051730 (*P* value = 0.04), rs8040868 (*P* value = 0.01) of CHRNA3, and rs16969968 (*P* value = 0.03) of CHRNA5. Additionally, a correlation was identified between rs3813567 of the CHRNB4 gene and the age at substance use onset (*P* value = 0.04).

**Conclusions:**

Variants in *CHRNA5*, *CHRNA3*, and *CHRNB4* may interact with SUD features that can influence the development and progression of the disorder among Jordanians.

**Supplementary Information:**

The online version contains supplementary material available at 10.1186/s12888-024-05898-7.

## Background

Substance use disorder (SUD) is characterized by the prolonged and frequent consumption of a wide variety of substances that necessitates both health care and treatment [1]. These substances include nicotine, alcohol, cannabinoids, opioids, depressants, stimulants, and hallucinogens [2]. The development of SUD depends on the duration of substance use; repeated use over a long period can lead to a chronic disorder known as addiction. Addiction refers to the condition where individuals experience physiological tolerance and withdrawal from the abused substance [3]. Health issues are the most critical outcomes of a substance use disorder, with poisoning and death being the primary consequences in addition to addiction [4]. Various diseases and illnesses can arise, including cancer onset and brain malfunction due to the impairment of certain areas in the brain that regulate vital body functions [5].

Even though environmental risk factors play a significant role in the development and progression of the disorder, it has been reported that genetic factors account for up to 70% of the risk of developing SUD [6]. However, like other chronic diseases, SUD is considered a complex illness where an individual’s environmental and genetic backgrounds interact to influence the disorder’s risk [7]. For example, being born to parents with a history of drug or alcohol use and being raised in areas with a high prevalence of substance use increases the risk of SUD for the children [8]. Conversely, certain environmental factors may act as protective parameters against substance use, such as social activities, parental monitoring, and restricted access to illegal substances [9]. Adolescence has been identified as a critical phase for controlling the disorder [10].

In Jordan, the pattern of substance use has significantly changed. While heroin consumption has dropped, the use of synthetic cannabinoids has drastically increased. The most commonly used substances among Jordanians were alcohol (39.8%) and synthetic cannabinoids (38.7%), compared to heroin usage (5.4%) [11]. The Anti-Narcotics Department in Jordan confirmed that substance use increased by 32% in 2019, with users predominantly between the ages of 18 and 22.

The nervous system comprises neuronal ion channels called neuronal nicotinic acetylcholine receptors (nAChRs) that bind to nicotine. These nAChRs are made up of α and β subunits encoded by nine α (*CHRNA2–CHRNA10*) and three β (*CHRNB2–CHRNB4*) genes. These subunits interact with dopaminergic and nicotinic neurons, influencing substance dependence by triggering the reward pathway [12, 13]. The genetic vulnerability of the nicotinic receptor gene cluster on chromosome 15, which includes *CHRNA5*, *CHRNA3*, and *CHRNB4*, to SUD has been extensively studied [14–16]. This cluster comprises various polymorphisms that manifest genetic risk for dependence on multiple substances [17]. CHRNA5 and CHRNA3 code for two nicotinic receptor subunits (α5 and α3, respectively), and the polymorphisms of both genes are found in high linkage disequilibrium [18]. It has been suggested that variants within both genes, *CHRNA5* and *CHRNA3*, influence the nicotine use pathways, leading to the development of nicotine addiction and a reduction in smoking cessation [19].

Aroche et al. (2020) identified a nominal association between homozygosity for major alleles of rs16969968 and rs588765 with an increased risk of crack addiction (GG, *P* = 0.032; CC, *P* = 0.036, respectively). Haplotype analyses showed significant associations (rs588765|rs16969968|rs514743 p global-corrected = 7.66 × 10^-5), highlighting a crucial role for rs16969968. These results support previous findings in cocaine addiction, aligning with the expected effects of cocaine on the cholinergic system and contrasting with significant GWAS findings related to nicotine addiction susceptibility [20]. Besson et al. (2019) demonstrated that rats carrying the α5SNP consumed more alcohol and exhibited increased relapse to alcohol-seeking post-abstinence. This heightened relapse was linked to altered insula activity, associated with interoception and observed via c-Fos immunostaining. The transgenic group also showed increased relapse to food seeking, with nicotine treatment reducing relapse in both transgenic and control rats. These results suggest that this human polymorphism affects reward processing and multiple addictions, not just smoking, indicating the potential for nicotinic receptor-targeted treatments for alcohol use and eating disorders [21]. Chmielowiec et al. (2022) identified significant haplotypes rs16969968, rs578776, and rs1051730, with G-T-T and G-C-T haplotypes unique to the study group, showing statistical significance (G-T-T: *p* = 0.01284; G-C-T: *p* = 0.00775 [22]. Cai et al. (2022) found associations between rs3743075, rs6495309 in CHRNA3, rs2304297 in CHRNA6, and rs1948 in CHRNB4 with sexual desire in heroin addiction patients. A haplotype block across CHRNA5, CHRNA3, and CHRNB4 was linked to changes in sexual desire post-long-term heroin use, highlighting the connection between nAChRs gene polymorphisms and heroin addiction phenotypes in the Chinese Han population [23]. Anton-Galindo et al. (2023) identified 68 DA and 27 5-HT genes associated with at least one GWAS on SUD or related behaviors. Six genes showed pleiotropic effects linked to at least three phenotypes: ADH1C, ARNTL, CHRNA3, HPRT1, HTR1B, and DRD2. Nominal associations were also found between DA gene sets and SUD, opioid use disorder, and other behaviors like irritability and neuroticism, as well as between the 5-HT-core gene set and neuroticism. Predicted gene expression correlates in the brain were identified for 19 DA or 5-HT genes, highlighting their role in addiction and related behaviors [24].

Moreover, Le Strat et al. (2020) provided strong evidence that specific genetic variants within the studied gene cluster (rs637137, rs3813567, and an ‘AGG’ haplotype) can differentiate adolescent tobacco dependence. These findings suggest the potential for using genetic markers of the CHRNA5/A3/B4 acetylcholine receptor gene cluster to identify adolescents at risk, enabling early interventions to prevent tobacco use disorder [25]. The most significant association with nicotine dependence has been reported for the rs16969968 polymorphism in the *CHRNA5* gene. This nonsynonymous SNP results in the replacement of aspartic acid with asparagine and has a bidirectional influence on substance use [26]. The minor allele G is associated with an increased risk for nicotine use and a decreased risk for cocaine use. This kind of association emerged due to the significant role of nAChRs in the dopaminergic reward system [12].

Various studies have explored the relationship between different genetic types and substance use disorders in Jordan. For example, research has focused on the Serotonin Transporter Gene (*SLC6A4/5-HTT*) [27, 28] and its polymorphic variant 5-HTTLPR, which have been implicated in regulating serotonin and associated with substance use behaviors. Other studies have examined the DRD4 exon III gene [29], which is linked to dopamine regulation and reward pathways, and the Mu opioid receptor gene (*OPRM1*) [30–32], known for its role in the body’s response to opioids and its potential influence on addiction susceptibility. This study aimed to investigate whether specific polymorphisms within three nicotinic receptor genes—*CHRNA5*, *CHRNA3*, and *CHRNB4*—are associated with an increased risk of substance use disorder among Jordanian males. These genes are fascinating because they encode subunits of nicotinic acetylcholine receptors, which are involved in neural signaling processes that can influence addictive behaviors. By examining these polymorphisms, we aim to contribute to the understanding of genetic factors that may confer risk for substance use disorders in this population, potentially providing insights that could lead to better prevention and treatment strategies.

## Materials and methods

### Recruitment and ascertainment

The samples consisted of unrelated cases and controls. A total of 495 Jordanian males were recruited from the National Centre for Rehabilitation of Addicts (NCRA) of the Ministry of Health in Jordan and the Drug Rehabilitation Centre of the Jordanian Public Security Directorate (DRC-PSD). The cohort was selected based on the substance use criteria outlined in the DSM-IV (APA, 2013). Inclusion criteria required participants to meet DSM-IV criteria for substance use disorder [33], be aged 18 years or older, provide written informed consent, and reside in Jordan. Exclusion criteria were designed to minimize confounding factors and included the presence of neurological diseases (e.g., epilepsy, Parkinson’s disease, multiple sclerosis), severe somatic diseases (e.g., significant cardiovascular disorders), severe psychiatric disorders (unless substance use disorder was primary), severe cognitive impairment or dementia, pregnancy, and current participation in another clinical study. This selection process ensured that the cohort accurately represented individuals with substance use disorder without the confounding influence of significant neurological, somatic, or psychiatric conditions.

Additionally, 497 healthy Jordanian males with no history of substance use or psychiatric disorders were chosen as controls. Subjects provided informed consent as approved by the Human Ethics Committee of the Jordanian Ministry of Health (MOH/REC/180,057), and the Institutional Review Board/Human Research Ethics Committee at Jordan University of Science and Technology (43/114/2018). This study was also approved by the Public Security Directorate (C/2/46/21,546) and King Abdullah University Hospital (43/114/2018).

According to the World Health Organization (WHO), 134,947 male adults in Jordan have substance use disorder, with a prevalence of 2.5% out of the country’s 9,531,712 total population (https://www.who.int/publications/m/item/jordan---who-special-initiative-for-mental-health*).* The sample size was determined using the OpenEpi program, version 3.01, with a 95% confidence interval. Considering a prevalence of 2.5% of substance use disorder in Jordan, a precision of 3%, and a design effect of 1, the sample size was calculated to be 105 participants. In our study, the sample size for the cases comprised 495 individuals. This number exceeds the requisite sample size.

### SNPs selection and genotyping

Genetic material was purified using venous blood samples in JUST laboratory and according to Wizard® Genomic DNA Purification Kit (Promega Corporation, USA). Agarose gel electrophoresis and the Nano-Drop ND-1000 UV-Vis Spectrophotometer (BioDrop, UK) detected the product for quality and quantity. A total of 15 SNPs in three nicotinic acetylcholine ACh receptor genes (*CHRNA5*, *CHRNA3*, and *CHRNB4*) were selected based on their biologically compelling association with substance use disorders among different ethnic groups. The GWAS studies on the *CHRNA5* gene suggest that genetic markers spanning this gene are implicated in the development of nicotine dependence [12]. This cluster confers genetic risk for dependence on various substances; therefore, it was a significant candidate for analyzing different substances. The sequencing technique was performed at the Australian Genome Research Facility (AGRF) (Australia) to genotype the selected single nucleotide polymorphisms SNPs within the three nicotinic receptor genes *CHRNA5*, *CHRNA3*, and *CRNB4*. The samples were genotyped using the Agena Bioscience MassARRAY® on a Compact Spectrometer, iPLEX GOLD chemistry.

### Statistical analysis

All SNPs were tested to fulfill the Hardy-Weinberg equilibrium (HWE) with an HWE-P value > 0.05. Additionally, minor allele frequencies were estimated using SNPStats software (2006 Institute Català d’Oncologia). The SNPStats software was also used to calculate genotypic and allelic frequencies, genetic associations, multiple genetic models, and genetic haplotype analyses. Pearson’s chi-square test and one-way ANOVA were used to analyze the genotype-phenotype relationship, while Odds Ratios (OR) with 95% confidence intervals (CI) were calculated. The Statistical Package for the Social Sciences (SPSS), version 25.0 (SPSS, Inc., Chicago, IL), was used to detect the relationship between different substance use disorder features and the investigated variants. A *p*-value less than 0.05 was considered to be statistically significant.

### Multiple testing corrections and an effective number of SNPs

Acadequateo Li and Ji (2005), the Bonferroni correction was used to set the significance cut-off at (α/n) where α = 0.05 and n number of tests [34]. Multiple testing correction sustains the overall *p*-value at a significance level of 0.025 or less. Moreover, adequate SNPs (Nem) were tested using Nyholt’s 2004 method [35]. Setting the confidence interval at 95% means that Zα/2 = 1.96. The maximum error rate will be considered 0.03.

## Results

### Characteristics of sample

In this study, all cohorts were Jordanian males of Arab descent who were hospitalized in 2018 for eight months at the National Centre for Rehabilitation of Addicts (NCRA) and the Drug Rehabilitation Centre of the Jordanian Public Security Directorate (DRC-PSD) in Jordan. The average age of the 495 cases was 28.6 ± 9.1, compared to 29 ± 6.9 for controls, with no statistically significant difference between cases and controls (*p*-value of approximately 0.437). The mean age of substance use onset was 24.3 ± 8.9, and the duration of substance use was 5.6 ± 5.1. The types of substances used make the cases vulnerable to SUD, including synthetic cannabinoids, cannabinoids, amphetamine, alcohol, benzodiazepines, opiates, cocaine, and cannabis. Remarkably, 88.2% of the cases were smokers, with synthetic cannabinoids being the most used substance (48%), while opiates and benzodiazepines were the most commonly used (4%). Smoking was the route of substance use for 70.9% of the cases, oral usage accounted for 15.2%, and injection was used by 4.3% of the subjects. The reasons for the cases to use one or more types of substances were mainly attributed to friends and other social risk factors such as circumstances or psychological insecurity; moreover, 30.0% of the cases used substances out of curiosity. On the other hand, the vast majority of the cases used only one substance (83.2%), 11.3% used two or more substances, while alcohol consumption alone accounted for 5.5%.

### Allelic frequency distribution and hardy weinberg equilibrium (HWE)

Chromosomal positions, minor alleles, and their frequencies in addition to the *p*-value of HWE for SNPs of (*CHRNA5*, *CHRNA3*, and *CHRNB4*) genes for cases and controls are summarized in Table 1. rs2869546, rs3743075, rs6495309 of *CHRNA5* and rs17408276 of *CHRNA3* were excluded from this study as they did not fulfill the HWE equation (P- value < 0.05).

**Table 1:** Minor allele frequencies among cases and healthy controls and the HWEc *P*-value of the candidate gene polymorphisms

Gene	SNP ID	SNP position^a^	Cases (*n* = 495)			Controls (*n* = 497)		
			MA^b^	MAF^c^	HWE^d^*P*-value	MA^b^	MAF^c^	HWE^d^*P*-value
CHRNA3	rs12914385	15:78606381	T	0.45	0.71	T	0.41	0.71
	rs2869546	15:78615003	C	0.26	0.04	C	0.27	0.09
	rs3743075	15:78617110	T	0.26	0.04	T	0.27	0.30
	rs6495307	15:78597979	T	0.28	0.18	T	0.30	0.13
	rs6495309	5:78622903	T	0.26	0.64	T	0.27	0.04
	rs1051730	15:78601997	A	0.42	0.78	A	0.40	1.0
	rs3743078	15:78602417	C	0.30	0.33	C	0.32	0.15
	rs8040868	15:78618839	C	0.48	0.32	C	0.46	0.65
CHRNA5	rs17408276	15:78589276	C	0.23	0.01	C	0.24	0.26
	rs621849	15:78580519	G	0.28	0.74	G	0.3	0.34
	rs637137	15:78581634	A	**0.26**	1.0	A	0.29	0.27
	rs684513	15:78566058	G	**0.24**	1.0	G	0.24	1.0
	rs951266	15:78586199	A	**0.42**	0.71	A	0.39	0.85
	rs16969968	15:78590583	A	**0.41**	0.64	A	0.39	0.7
CHRNB4	rs3813567	15:78642209	G	**0.27**	0.42	G	0.28	0.38

^a^Chromosome positions are based on NCBI Human Genome Assembly Build.^b^MA: minor allele. ^c^MAF: minor allele frequency. ^d^HWE: Hardy—Weinberg equilibrium

### Genetic association analyses outcome

Table 2 illustrates the genetic correlation between the investigated polymorphisms and SUD. Allelic and genotypic frequencies among cases and controls for each SNP have also been estimated. As Table 2 shows, there was no significant difference in the frequency distribution for any SNP between cases and controls. Furthermore, the *P*-value and Chi-square were used to identify significant associations. In this regard, none of the tested alleles or genotypes were found to be correlated with SUD.

**Table 2:** Genetic association between the polymorphisms and SUD

Gene	SNP ID	Allelic and Genotypic Frequencies in Cases and Controls				
		Allele/ Genotype	Cases **(** ***n*** ** = 495)**	Controls **(** *n* ** = 497)**	*P*-value*	Chi-square
*CHRNA3*	rs12914385	C	544(0.55)	588(0.59)	0.07	3.2
		T	438(0.45)	402(0.41)		
		CC	153(0.31)	172(0.35)	0.16	3.6
		CT	238(0.48)	244(0.49)		
		TT	100(0.2)	79(0.16)		
	rs6495307	C	709(0.72)	695(0.70)	0.48	0.48
		T	277(0.28)	291(0.30)		
		CC	261(0.53)	252(0.51)	0.79	0.46
		CT	187(0.38)	191(0.39)		
		TT	45(0.09)	50(0.10)		
	rs1051730	G	574(0.58)	598(0.6)	0.34	0.87
		A	410(0.42)	392(0.40)		
		GG	169(0.34)	180(0.36)	0.62	0.95
		GA	236(0.48)	238(0.48)		
		AA	87(0.18)	77(0.16)		
	rs3743078	G	672(0.7)	673(0.68)	0.40	0.68
		C	292(0.30)	317(0.32)		
		GG	239(0.50)	236(0.48)	0.69	0.72
		GC	194(0.40)	201(0.41)		
		CC	49(0.10)	58(0.12)		
	rs8040868	T	509(0.52)	535(0.54)	0.30	1.06
		C	471(0.48)	451(0.46)		
		TT	138(0.28)	148(0.30)	0.55	1.16
		CT	233(0.48)	239(0.48)		
		CC	119(0.24)	106(0.22)		
*CHRNA5*	rs621849	A	699(0.72)	689(0.70)	0.28	1.12
		G	273(0.28)	299(0.30)		
		AA	253(0.52)	245(0.50)	0.53	1.26
		GA	193(0.40)	199(0.40)		
		GG	40(0.08)	50(0.10)		
	rs637137	T	687(0.74)	695(0.71)	0.14	2.10
		A	241(0.26)	283(0.29)		
		TT	254(0.55)	252(0.52)	0.26	2.66
		TA	179(0.39)	191(0.39)		
		AA	31(0.07)	46(0.09)		
	rs684513	C	751(0.76)	752(0.76)	0.91	0.01
		G	231(0.24)	234(0.24)		
		CC	287(0.58)	287(0.58)	0.99	0.01
		CG	177(0.36)	178(0.36)		
		GG	27(0.05)	28(0.06)		
	rs951266	G	571(0.58)	595(0.61)	0.20	1.59
		A	417(0.42)	387(0.39)		
		GG	167(0.34)	179(0.36)	0.41	1.77
		GA	237(0.48)	237(0.48)		
		AA	90(0.18)	75(0.15)		
	rs16969968	A	403(0.41)	381(0.39)	0.22	1.52
		G	573(0.59)	607(0.61)		
		AA	86(0.18)	71(0.14)	0.16	2.01
		AG	231(0.47)	239(0.48)		
		GG	171(0.35)	184(0.37)		
*CHRNB4*	rs3813567	A	723(0.73)	707(0.72)	0.34	0.90
		G	261(0.27)	281(0.28)		
		AA	269(0.55)	257(0.52)	0.64	0.87
		AG	185(0.38)	193(0.39)		
		GG	38(0.08)	44(0.09)		

* *P*- Value < 0.025 was considered as significant after performing Bonferroni correction

Other genetic models were used further to investigate the association between the SNPs and SUD. Table 3 presents two additional genetic models (dominant and recessive) and the dominant model in Table 2. The recessive genetic model refers to a pattern of inheritance where a trait or characteristic is expressed only when an individual carries two copies of the recessive allele, one inherited from each parent. In this model, individuals with one copy of the recessive allele may not exhibit the trait themselves, as the dominant allele suppresses its expression. However, carriers of one copy of the recessive allele can pass it on to their offspring. When two carriers carrying one copy of the recessive allele have offspring together, there is a chance that their offspring could inherit two copies of the recessive allele, resulting in the expression of the trait. The dominant model tests for homozygous dominant vs. heterozygous/homozygous recessive, and the recessive model tests for homozygous dominant/heterozygous vs. homozygous recessive. As the statistical analysis in Table 3 demonstrates, none of the studied SNPs show any influence on SUD risk using the dominant and recessive models.

**Table 3:** Different genetic models analysis between the investigated SNPs and SUD

Gene	SNP ID	Model	Genotype	Cases (%)	Controls (%)	OR (95% CI)	*P*-Value
***CHRNA3***	rs12914385	Dominant	C/C C/T-T/T	153 (31.2%) 338 (68.8%)	172 (34.8%) 153 (31.2%)	1.00 0.85 (0.65–1.11)	0.23
		Recessive	C/C-C/T T/T	391 (79.6%) 100 (20.4%)	416 (84%) 79 (16%)	1.00 1.35 (0.97–1.87)	0.07
	rs6495307	Dominant	C/C C/T-T/T	261 (52.9%) 232 (47.1%)	252 (51.1%) 241 (48.9%)	1.00 1.08 (0.84–1.38)	0.57
		Recessive	C/C-C/T T/T	448 (90.9%) 45 (9.1%)	443 (89.9%) 50 (10.1%)	1.00 1.12 (0.74–1.72)	0.59
	rs1051730	Dominant	G/G G/A-A/A	169 (34.4%) 323 (65.7%)	180 (36.4%) 315 (63.6%)	1.00 0.92 (0.71–1.19)	0.51
		Recessive	G/G-G/A A/A	405 (82.3%) 87 (17.7%)	418 (84.4%) 77 (15.6%)	1.00 0.86 (0.61–1.20)	0.37
	rs3743078	Dominant	G/G C/G-C/C	239 (49.6%) 243 (50.4%)	236 (47.7%) 259 (52.3%)	1.00 1.08 (0.84–1.39)	0.55
		Recessive	G/G-C/G C/C	433 (89.8%) 49 (10.2%)	437 (88.3%) 58 (11.7%)	1.00 1.17 (0.78–1.75)	0.44
	rs8040868	Dominant	T/T C/T-C/C	138 (28.2%) 352 (71.8%)	148 (30%) 345 (70%)	1.00 0.91 (0.69–1.20)	0.52
		Recessive	T/T-C/T C/C	371 (75.7%) 119 (24.3%)	387 (78.5%) 106 (21.5%)	1.00 0.85 (0.63–1.15)	0.30
***CHRNA5***	rs621849	Dominant	A/A G/A-G/G	253 (52.1%) 233 (47.9%)	245 (49.6%) 249 (50.4%)	1.00 1.10 (0.86–1.42)	0.44
		Recessive	A/A-G/A G/G	446 (91.8%) 40 (8.2%)	444 (89.9%) 50 (10.1%)	1.00 1.26 (0.81–1.94)	0.30
	rs637137	Dominant	T/T A/T-A/A	254 (54.7%) 210 (45.3%)	252 (51.5%) 237 (48.5%)	1.00 1.14 (0.88–1.47)	0.32
		Recessive	T/T-A/T A/A	433 (93.3%) 31 (6.7%)	443 (90.6%) 46 (9.4%)	1.00 1.45 (0.90–2.33)	0.12
	rs684513	Dominant	C/C C/G-G/G	287 (58.5%) 204 (41.5%)	287 (58.2%) 206 (41.8%)	1.00 1.01 (0.78–1.30)	0.94
		Recessive	C/C-C/G G/G	464 (94.5%) 27 (5.5%)	465 (94.3%) 28 (5.7%)	1.00 1.03 (0.60–1.78)	0.90
	rs951266	Dominant	G/G G/A-A/A	167 (33.8%) 327 (66.2%)	179 (36.5%) 312 (63.5%)	1.00 0.89 (0.69–1.16)	0.38
		Recessive	G/G-G/A A/A	404 (81.8%) 90 (18.2%)	416 (84.7%) 75 (15.3%)	1.00 0.81 (0.58–1.13)	0.22
	rs16969968	Dominant	G/G G/A-A/A	171 (35%) 317 (65%)	184 (37.2%) 310 (62.8%)	1.00 0.91 (0.70–1.18)	0.47
		Recessive	G/G-G/A A/A	402 (82.4%) 86 (17.6%)	423 (85.6%) 71 (14.4%)	1.00 0.78 (0.56–1.11)	0.16
*CHRNB4*	rs3813567	Dominant	A/A G/A-G/G	269 (54.7%) 223 (45.3%)	257 (52%) 237 (48%)	1.00 1.11 (0.87–1.43)	0.40
		Recessive	A/A-G/A G/G	454 (92.3%) 38 (7.7%)	450 (91.1%) 44 (8.9%)	1.00 1.17 (0.74–1.84)	0.50

* *P*- Value < 0.025 was considered as significant after performing Bonferroni correction

OD: odd ratio

CI: Confidence interval

### The association between haplotypes of studied genes and SUD

The association between haplotypes of the studied genes and substance use disorder (SUD) was thoroughly analyzed, with the findings presented in Table 4. This analysis involved examining specific regions of the *CHRNA3* and *CHRNA5* genes to identify patterns of single nucleotide polymorphisms (SNPs) that might be linked to SUD. For the *CHRNA3* gene, a block of seven haplotypes was identified, comprising the SNPs rs12914385, rs6495307, rs1051730, rs3743078, and rs8040868. Similarly, for the *CHRNA5* gene, a block of four haplotypes was identified, consisting of the SNPs rs621849, rs637137, rs684513, rs951266, and rs16969968. These haplotypes represent different combinations of genetic variants tested for their potential association with SUD. Despite the thorough analysis, the statistical tests indicated no significant associations between these haplotypes and SUD. The *p*-values for the associations were all greater than 0.05, suggesting that the variations in these haplotypes do not have a meaningful correlation with the likelihood of developing SUD. Therefore, based on this study, the haplotypes within the *CHRNA3* and *CHRNA5* genes do not appear to be linked to an increased or decreased risk of substance use disorder.

**Table 4:** Haplotypes association with SUD

Haplotype CHRNA3 Block (rs12914385, rs6495307, rs1051730, rs3743078, rs8040868)	Frequency of block	Frequency		Cumulative frequency	Odds ratio (95%) CI	*P*-value
		case	control			
TCAGC	0.39	0.408	0.3846	0.3963	1	NA
CCGCT	0.26	0.2646	0.2735	0.6647	1.10 (0.88–1.37)	0.40
CTGGT	0.24	0.2309	0.2497	0.9047	1.14 (0.91–1.43)	0.25
CCGCC	0.02	0.0215	0.03	0.9312	1.42 (0.79–2.56)	0.24
CTGGC	0.02	0.0218	0.0253	0.9548	1.28 (0.68–2.40)	0.45
TTGGC	0.01	0.0172	0.0089	0.9679	0.53 (0.23–1.25)	0.15
TCGCT	0.01	0.0112	0.0086	0.9782	0.81 (0.34–1.96)	0.65
Haplotype *CHRNA5* Block (rs621849, rs637137, rs684513, rs951266, rs16969968)						
ATCAA	0.39	0.407	0.3799	0.3933	1	NA
GTCGG	0.28	0.2713	0.2963	0.6769	1.16 (0.93–1.44)	0.19
AAGGG	0.22	0.2242	0.2256	0.9023	1.09 (0.86–1.37)	0.49
AACGG	0.05	0.0487	0.0623	0.9581	1.36 (0.91–2.03)	0.14
ATCGG	0.01	0.0224	0.0131	0.9755	0.66 (0.33–1.31)	0.23

* *P-Value < 0.05 considered as significant

### The association between the studied genes and nicotine dependence

On the other hand, nicotine dependence was examined in this study. To define the underlying role of SNPs in three nicotinic ACh receptor genes (*CHRNA5*, *CHRNA3*, and *CHRNB4*) in the risk of nicotine use alone, genetic analysis of a cohort of nicotine users among substance users was performed, as illustrated in Table 5. Slight differences were observed in the allelic and genotypic frequencies between nicotine users and controls, as shown in the table. Additionally, genetic models were used to explore the association between SNPs and nicotine dependence. Table 6 presents the two genetic models (dominant and recessive). As indicated in Table 6, none of the studied SNPs show any effect on nicotine dependence when using the dominant and recessive models.

**Table 5:** Genetic association between the polymorphisms and nicotine dependence

Gene	SNP ID	Allelic and Genotypic Frequencies in Cases and Controls				
		Allele/ Genotype	Nicotine use ***n*** ** = 427 (%)**	Controls ***n*** ** = 497 (%)**	*P*-value*	Chi-square
CHRNA3	rs12914385	C	461(0.55)	588(0.59)	0.06	3.56
		T	377(0.45)	402(0.41)		
		CC	127(0.3)	172(0.35)	0.053	3.73
		CT	207(0.49)	244(0.49)		
		TT	85(0.2)	79(0.16)		
	rs6495307	C	606(0.72)	695(0.70)	0.48	0.48
		T	236(0.28)	291(0.30)		
		CC	224(0.53)	252(0.51)	0.49	0.45
		CT	158(0.38)	191(0.39)		
		TT	39(0.09)	50(0.10)		
	rs1051730	G	489(0.58)	598(0.6)	0.34	0.9
		A	351(0.42)	392(0.40)		
		GG	142(0.34)	180(0.36)	0.34	0.9
		GA	205(0.49)	238(0.48)		
		AA	73(0.17)	77(0.16)		
	rs3743078	G	575(0.70)	673(0.68)	0.4	0.68
		C	249(0.30)	317(0.32)		
		GG	204(0.50)	236(0.48)	0.37	0.79
		GC	167(0.41)	201(0.41)		
		CC	41(0.10)	58(0.12)		
	rs8040868	T	431(0.52)	535(0.54)	0.24	1.32
		C	405(0.48)	451(0.46)		
		TT	116(0.28)	148(0.30)	0.23	1.40
		CT	199(0.48)	239(0.48)		
		CC	103(0.25)	106(0.22)		
CHRNA5	rs621849	A	598(0.72)	689(0.70)	0.24	1.34
		G	230(0.28)	299(0.30)		
		AA	217(0.52)	245(0.50)	0.2	1.51
		GA	164(0.40)	199(0.40)		
		GG	33(0.08)	50(0.10)		
	rs637137	T	591(0.74)	695(0.71)	0.11	2.49
		A	203(0.26)	283(0.29)		
		TT	217(0.55)	252(0.52)	0.043	4.09
		TA	157(0.4)	191(0.39)		
		AA	23(0.06)	46(0.09)		
	rs684513	C	643(0.77)	752(0.76)	0.81	0.05
		G	195(0.23)	234(0.24)		
		CC	245(0.58)	287(0.58)	0.65	0.20
		CG	153(0.37)	178(0.36)		
		GG	21(0.05)	28(0.06)		
	rs951266	G	485(0.57)	595(0.61)	0.17	1.83
		A	359(0.42)	387(0.39)		
		GG	140(0.33)	179(0.36)	0.16	1.90
		GA	205(0.49)	237(0.48)		
		AA	77(0.18)	75(0.15)		
	rs16969968	A	347(0.42)	381(0.39)	**0.17**	1.86
		G	485(0.58)	607(0.61)		
		AA	74(0.18)	71(0.14)	0.13	2.18
		AG	199(0.48)	239(0.48)		
		GG	143(0.34)	184(0.37)		
CHRNB4	rs3813567	A	619(0.74)	707(0.72)	0.35	0.87
		G	223(0.26)	281(0.28)		
		AA	230(0.55)	257(0.52)	0.35	0.85
		AG	159(0.38)	193(0.39)		
		GG	32(0.08)	44(0.09)		

* *P*- Value < 0.025 was considered as significant after performing Bonferroni correction

**Table 6:** Different genetic models analysis between the investigated SNPs and nicotine dependence

Gene	SNP ID	Model	Genotype	Cases (%)	Controls (%)	OR (95% CI)	*P*-Value
***CHRNA3***	rs12914385	Dominant	C/C C/T-T/T	127 (30.3%) 292 (69.7%)	172 (34.8%) 323 (65.2%)	1.00 0.82 (0.62–1.08)	0.15
		Recessive	C/C-C/T T/T	334 (79.7%) 85 (20.3%)	416 (84%) 79 (16%)	1.00 0.75 (0.53–1.05)	0.09
	rs6495307	Dominant	C/C C/T-T/T	224 (53.2%) 197 (46.8%)	252 (51.1%) 241 (48.9%)	1.00 1.08 (0.84–1.38)	0.57
		Recessive	C/C-C/T T/T	382 (90.7%) 39 (9.3%)	443 (89.9%) 50 (10.1%)	1.00 1.11 (0.71–1.72)	0.65
	rs1051730	Dominant	G/G G/A-A/A	142 (33.8%) 378 (66.2%)	180 (36.4%) 315 (63.6%)	1.00 0.89 (0.68–1.17)	0.42
		Recessive	G/G-G/A A/A	347 (82.6%) 73 (17.4%)	418 (84.4%) 77 (15.6%)	1.00 0.88 (0.62–1.24)	0.46
	rs3743078	Dominant	G/G C/G-C/C	204 (49.5%) 208 (50.5%)	236 (47.7%) 259 (52.3%)	1.00 1.08 (0.83–1.40)	0.58
		Recessive	G/G-C/G C/C	371 (90%) 41 (9.9%)	437 (88.3%) 58 (11.7%)	1.00 1.20 (0.79–1.83)	0.39
	rs8040868	Dominant	T/T C/T-C/C	116 (27.8%) 302 (72.2%)	148 (30%) 345 (70%)	1.00 0.90 (0.67–1.19)	0.45
		Recessive	T/T-C/T C/C	315 (75.4%) 103 (24.6%)	387 (78.5%) 106 (21.5%)	1.00 0.84 (0.61–1.14)	0.26
***CHRNA5***	rs621849	Dominant	A/A G/A-G/G	217 (52.4%) 197 (47.6%)	245 (49.6%) 249 (50.4%)	1.00 1.12 (0.86–1.45)	0.4
		Recessive	A/A-G/A G/G	381 (92%) 33 (8%)	444 (89.9%) 50 (10.1%)	1.00 1.30 (0.82–2.06)	0.26
	rs637137	Dominant	T/T A/T-A/A	217 (54.7%) 180 (45.3%)	252 (51.5%) 237 (48.5%)	1.00 1.13 (0.87–1.48)	0.35
		Recessive	T/T-A/T A/A	374 (94.2%) 23 (5.8%)	443 (90.6%) 46 (9.4%)	1.00 1.69 (1.00-2.84)	0.04
	rs684513	Dominant	C/C C/G-G/G	245 (58.5%) 174 (41.5%)	287 (58.2%) 206 (41.8%)	1.00 1.01 (0.78–1.32)	0.94
		Recessive	C/C-C/G G/G	398 (95%) 21 (5%)	465 (94.3%) 28 (5.7%)	1.00 1.03 (0.60–1.78)	0.66
	rs951266	Dominant	G/G G/A-A/A	140 (33.2%) 282 (66.8%)	179 (36.5%) 312 (63.5%)	1.00 0.87 (0.66–1.14)	0.3
		Recessive	G/G-G/A A/A	345 (81.8%) 77 (18.2%)	416 (84.7%) 75 (15.3%)	1.00 0.81 (0.57–1.14)	0.23
	rs16969968	Dominant	G/G G/A-A/A	143 (34.4%) 273 (65.6%)	184 (37.2%) 310 (62.8%)	1.00 0.88 (0.67–1.16)	0.37
		Recessive	G/G-G/A A/A	342 (82.2%) 74 (17.8%)	423 (85.6%) 71 (14.4%)	1.00 0.78 (0.56–1.11)	0.16
*CHRNB4*	rs3813567	Dominant	A/A G/A-G/G	230 (54.6%) 191 (45.4%)	257 (52%) 237 (48%)	1.00 1.11 (0.86–1.44)	0.43
		Recessive	A/A-G/A G/G	389 (92.4%) 32 (7.6%)	450 (91.1%) 44 (8.9%)	1.00 1.19 (0.74–1.91)	0.47

*P*- Value < 0.025 was considered as significant after performing Bonferroni correction

OD: odd ratio

CI: Confidence interval

### SUD features and nicotinic ACh receptor genes

It is well known that certain environmental factors can act as protective or risk factors for SUD. In this regard, several disorder characteristics (Table 7) were considered in this study. Table 7 summarizes these features and describes the relationship between SUD features and CHRNA5, CHRNA3, and CHRNB4 SNPs. Our findings suggest that the route of substance use was associated with rs1051730 (*P* value = 0.04) and rs8040868 (*P* value = 0.01) of CHRNA3, as well as rs16969968 (*P* value = 0.03) of CHRNA5. Additionally, the results revealed a correlation between rs3813567 of the *CHRNB4* gene and the age at substance use onset (*P* value = 0.04). Although the difference in mean age between the three genotypes of rs3813567 was slight, cases with the variant genotype (GG) tended to start using substances at an earlier age, with an average age of 22, compared to cases with the wild type (AA), who had a mean age of 25 years. This finding suggests that the rs3813567 SNP may be involved in the onset of substance use among individuals with the variant genotype.

**Table 7:** Association between several SUD features and three nicotinic ACh receptor gene variants

CHRNA3 Polymorphisms	Addiction features							
	Age of cases*	Age at onset*	Duration of substance use (years) *	Motives for substance use**	Types of substances**	Smoking**	Route of substance use**	Number of substances**
rs12914385	0.642	0.242	0.903	0.665	0.505	0.541	0.066	0.748
rs6495307	0.654	0.300	0.179	0.231	0.778	0.112	0.569	0.499
rs1051730	0.746	0.393	0.885	0.545	0.379	0.424	**0.040**	0.303
rs3743078	0.526	0.437	0.435	0.405	0.696	0.728	0.327	0.902
rs8040868	0.306	0.208	0.822	0.573	0.213	0.583	**0.013**	0.586
***CHRNA5*** **Polymorphisms**								
rs621849	0.713	0.365	0.319	0.521	0.787	0.296	0.686	0.625
rs637137	0.845	0.508	0.260	0.377	0.422	0.505	0.173	0.770
rs684513	0.839	0.165	0.098	0.612	0.641	0.811	0.195	0.494
rs951266	0.541	0.277	0.813	0.613	0.343	0.598	0.058	0.310
rs16969968	0.736	0.174	0.641	0.629	0.428	0.722	**0.031**	0.300
**CHRNB4** **Polymorphism**								
rs3813567	0.122	**0.044**	0.651	0.425	0.911	0.168	0.712	0.918

* Analysis of variance (ANOVA) was used to determine the association

**Pearson’s chi-squared test was used to determine the association

## Discussion

Substance Use Disorder (SUD) is a general term used to describe several conditions involving the use of legal and illicit substances that lead to clinical impairment, mental instability, and the devastation of an individual’s social life. According to the Diagnostic and Statistical Manual of Mental Disorders V (DSM-V) 2013, SUDs include substance abuse, dependence, and addiction [36]. SUD is a worldwide burden, and it has become a severe concern among Arabs, including Jordanians [37]. SUDs are considered complex disorders in which both genetic and environmental factors are responsible for their development. Combining these factors can decrease the risk of developing SUD and constrain its progression [36]. Studies have demonstrated that genetic variants within significant genes contribute to SUD risk, including genes that code for aldehyde dehydrogenases (ALDH), solute carriers (SLC), gamma-aminobutyric acid (GABAA) receptors, cytochromes P450 (CYPs), dopamine receptor D (DRD), and opioid receptors (OPR) [38]. This study examined 15 SNPs within the nicotinic receptor subunit gene cluster (*CHRNA5*, *CHRNA3*, and *CHRNB4*). These genes have a strong association with an increased risk of nicotine use [39].

nAChRs are ligand-gated channels triggered by exogenous agonists, including nicotine or tobacco-specific nitrosamines. They are mainly expressed in the brain and play a key role in addiction pathways. They are also described in other cell types, mediating several biological actions via intracellular calcium influx. However, since nAChRs are involved in a signaling pathway that elucidates a significant association between SNPs spanning the 15q24 region and nicotine dependence, polymorphisms within that region are considered critical candidate risk factors for SUD, particularly nicotine dependence [40]. The link between SNPs in nicotinic ACh receptor genes and SUD has shown inconsistency among various populations. The current study investigated substances in correlation with the assigned SNPs. Moreover, nicotine was uniquely studied for its association with nicotinic ACh receptor gene SNPs among Jordanian males. However, the performed genetic association analyses revealed no correlation between CHRNA5, CHRNA3, and CHRNB4 and SUD SNPs.

In contrast with other studies, rs16969968 CHRNA5 SNP was not in correlation with nicotine dependence [41–44]. The variation in the association among different populations could be attributed to the significant impact of certain variants on specific populations. The minor allele frequency of rs16969968 varies between populations; in Africa, it was estimated to be near 0%, while it was up to 37% in Europeans. However, this study assessed minor allele frequency at 28% [42]. The rs16969968 variant modulates the α5 subunit, resulting in a depletion in nicotine receptor function [45], which supports the hypothesis that the reduced function of the nicotinic receptor is linked to an elevated risk for nicotine dependence [12]. Another SNP within *CHRNA5* (rs588765) was also implicated in nicotine dependence by manipulating the expression of *CHRNA5* [12]. In addition to nicotine, rs684513 of *CHRNA5* was reported as a risk marker for cocaine dependence in African-Americans [46].

Although genetic susceptibility to SUD serves as a baseline for the biological risk factor, it should be connected to the demographic and clinical features of SUD to enhance the treatment protocol and prevent the onset of the disorder. In this regard, we analyzed several SUD features and their association with the SNPs of *CHRNA5*, *CHRNA3*, and *CHRNB4*. Our findings suggest that rs1051730 and rs8040868 of *CHRNA3 and rs16969968 of CHRNA5* were somehow linked to the route of substance use. Additionally, a connection between rs3813567 of the *CHRNB4* gene and the age at substance use onset was speculated, which alludes to the involvement of the rs3813567 SNP in the early onset of substance use among cases carrying the corresponding SNP. However, few studies have explored the association between SUD and genetic variants. Within *CHRNA3*, the rs8023462 variant was associated with the age of onset for nicotine and alcohol use [47], while in a European population, rs1051730 was related to smoking quantity [48].

Several limitations may be encountered when investigating the genetic association of substance use disorders (SUDs) in Jordan. Firstly, while a representative sample of cases with SUD should be incorporated, factors such as sociocultural influences and local variation in genetic predisposition may significantly influence substance use behaviors, thus limiting the study. Secondly, self-reported data for clinical characteristics and medical history increase the possibility of bias and elevate the frequency of misclassification errors. Thirdly, the susceptibility to SUD caused by the limited set of genes studied in the current research could be considered a small part of the complete picture of all genetic causes, considering the complexity of SUD as a multifactorial disorder. Other genetic and environmental factors, as well as gene-environment interactions, are more likely to participate in the development and progression of SUD. These reasons emphasize the need for additional diagnostic research exploring the complete genetic condition of this disorder. More detailed studies with larger samples are warranted to investigate the dynamic associations between genes and drug use behaviors over time. Finally, to emphasize the observed associations in genetic association studies, they must be replicated several times in independent groups. In conclusion, while the current findings provide helpful insight into the genetic basis of SUD in the Jordanian population, it is crucial to interpret them in light of these limitations.

## Conclusion

In summary, no significant association was found between genetic variants within the three nicotinic ACh receptor genes (*CHRNA5*, *CHRNA3*,* CHRNB4*) and SUD when analyzing a broad range of substance together. However, variants in *CHRNA5* and *CHRNA3* may interact with specific features of SUD, potentially influencing the development and progression of the disorder. The complexity of SUD hinders research progress in this field. As only a few genetic association research [49–51] have been conducted in Jordan, more studies focusing on both genetic and non-genetic factors are needed to improve the assessment of SUD and to provide suitable healthcare for affected individuals. Additionally, studies should be conducted with more precise criteria. Further research is also necessary to elucidate the role of genetic variants in dependence on specific substance types.

### Electronic supplementary material

Below is the link to the electronic supplementary material.

**Supplementary Material 1:** The complete processed SNP genotypic data for the three genes *CHRNA5*, *CHRNA3*, and *CHRNB4*

## Data Availability

The complete processed SNP genotypic data for the three genes CHRNA5, CHRNA3, and CHRNB4 is available as a supplementary file.

## Abbreviations

SUD: Substance use disorder
nAChRs: Neuronal nicotinic acetylcholine receptors
*OPRM1*: Mu opioid receptor
NCRA: National Centre for Rehabilitation of Addicts
DRC-PSD: Drug Rehabilitation Centre of the Jordanian Public Security Directorate
DSM-: Manual of Mental Disorders
AGRF: Australian Genome Research Facility
SNPs: Single nucleotide polymorphisms
HWE: Hardy-Weinberg equilibrium
SPSS: Statistical Package for the Social Sciences
ALDH: Aldehyde dehydrogenases
SLC: Solute carriers
GABAA: ƴ-aminobutyric acid
CYPs: Cytochromes P450
DRD: Dopamine receptor D
OPR: Opioid Receptors
ACh: Acetylcholine
